# Impact of a machine learning algorithm on time to palliative care in a primary care population: protocol for a stepped-wedge pragmatic randomized trial

**DOI:** 10.1186/s12904-022-01113-0

**Published:** 2023-02-03

**Authors:** Ethan P. Heinzen, Patrick M. Wilson, Curtis B. Storlie, Gabriel O. Demuth, Shusaku W. Asai, Gavin M. Schaeferle, Mairead M. Bartley, Rachel D. Havyer

**Affiliations:** 1 Robert D. and Patricia E. Kern Center for the Science of Healthcare Delivery, Mayo Clinic, Rochester, MN USA; 2 Community Internal Medicine, Mayo Clinic, Rochester, MN USA

**Keywords:** Randomized trial, Protocol, Machine learning

## Abstract

**Background:**

As primary care populations age, timely identification of palliative care need is becoming increasingly relevant. Previous studies have targeted particular patient populations with life-limiting disease, but few have focused on patients in a primary care setting. Toward this end, we propose a stepped-wedge pragmatic randomized trial whereby a machine learning algorithm identifies patients empaneled to primary care units at Mayo Clinic (Rochester, Minnesota, United States) with high likelihood of palliative care need.

**Methods:**

42 care team units in 9 clusters were randomized to 7 wedges, each lasting 42 days. For care teams in treatment wedges, palliative care specialists review identified patients, making recommendations to primary care providers when appropriate. Care teams in control wedges receive palliative care under the standard of care.

**Discussion:**

This pragmatic trial therefore integrates machine learning into clinical decision making, instead of simply reporting theoretical predictive performance. Such integration has the possibility to decrease time to palliative care, improving patient quality of life and symptom burden.

**Trial registration:**

Clinicaltrials.gov NCT04604457, restrospectively registered 10/26/2020.

**Protocol:**

v0.5, dated 9/23/2020

## Background

Palliative care aims to improve quality of life, decrease symptom burden, and guide value-concordant care for patients dealing with serious illness. Early integration of palliative care has been shown to improve quality of life (QOL) and mood for patients and decrease health care resource utilization [1–5]. Involvement of palliative care is increasingly important at a population level with the increasing age and medical complexity of populations. Therefore, timely identification of palliative care need is critical. Such identification of patients with a need for palliative care can be difficult, however, at a primary care population level. The use of artificial intelligence has been postulated as a way to help with timely identification of patients [6].

However, integration of machine learning algorithms into clinical practice proves to be more difficult than simply reporting theoretical performance. Previous studies suggest that even among patients predicted by an algorithm to have high palliative care need, providers may believe that palliative care needs are negligible or already being met [7]. Other studies suggest that engagement of primary care providers, patients, and caregivers might be difficult, in some cases because palliative care is misunderstood or negatively perceived [8–10]. Therefore, performance of an algorithm is likely to be lower in practice than it would be in a hold-out set in model development. Pragmatic trials are a meaningful mechanism to examine the real-world impact of algorithms on clinical decision-making. Courtright et al applies the pragmatic trial framework in the context of palliative care, but offers insight only into the inpatient population [7]. There is need, therefore, for an outpatient-focused pragmatic clinical trial to examine the effect of machine learning on referral to palliative care.

The primary aim of this study is to assess the impact of the integration of a machine learning algorithm into clinical workflow within a primary care population. Our hypothesis is that the integration of the algorithm will reduce the time to palliative care consultation for patients in the primary care outpatient setting.

## Methods/Design

### Setting

Mayo Clinic (Rochester, MN, United States) is an academic medical center with a community primary care population of approximately 119,000 adult patients, consisting of 42 care teams nested within 9 care team clusters across 5 different sites.

### Algorithm development

Adult patients assigned to a primary care unit from October 1, 2018, to December 31, 2019, were included; patients under 18 were excluded. Hospice and death were considered censoring events, and patients were removed from the data for 75 days following a palliative care consult. Historical data from the primary care population were obtained, yielding 116108 unique patients and 571 palliative care consults. A time-to-event Poisson gradient-boosting machine (GBM) with time-dependent covariates was trained to predict presence of a palliative care consult. To reduce the overall training time, model selection and training were done on a subset of the training data, with a 10-1 control-case ratio. Nested cross-validation was used to optimize tuning parameters. Covariates included age, sex, lab values, diagnosis codes, clinical note metadata, opioid usage, pain scores, prior inpatient utilization, and time since last appointment. The mean training survival concordance (c-statistic) obtained in cross-validation was 0.96.

### Trial design

To determine the impact of the algorithm on time to needed palliative care, a stepped-wedge pragmatic randomized clinical trial will evaluate the integration of the algorithm into the primary care practice. The process of integrating the algorithm use into the primary care setting was developed with input from generalists. Adult patients empaneled to a primary care unit from July 2020 to June 2021 will be included in this study. Patients recently seen by palliative care within the previous 75 days, patients under the age of 18, currently enrolled in hospice or residing in skilled nursing facility at the time of the algorithm refreshing will be excluded.

**Fig. 1:**
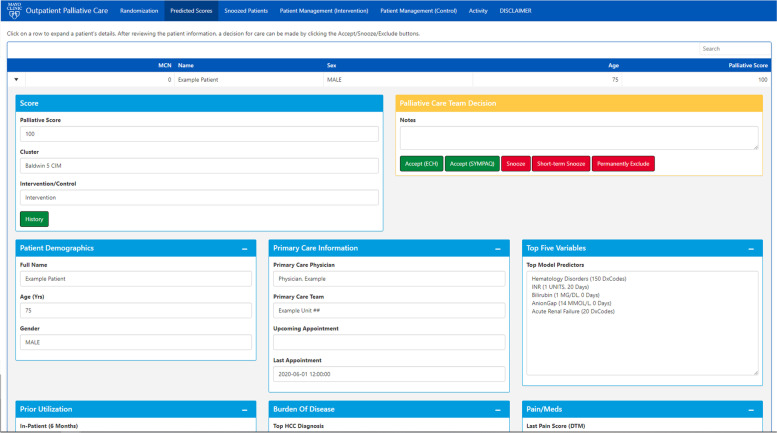
A custom user interface to present the top 40 patients as predicted by the algorithm

In this study, the algorithm will be run on current patient data weekly for all eligible primary care patients. The 40 patients with the highest predicted palliative care score will be presented to the investigator palliative medicine specialists [RH, MB] alongside relevant clinical information in a custom user interface designed using R Shiny (Fig. 1).

Weekly, the palliative care specialists will review the presented patients and make determinations on the appropriateness of palliative care referral, performing chart review if necessary to find patient characteristics that may aid decision-making. The palliative care specialist will then have the option to “snooze” a patient (reject them for 90 days), “short snooze” (review again in 2 weeks) or “accept” a patient (determine that palliative care referral is recommended for the patient). For accepted patients in the treatment arm, the palliative care specialist will reach out to the patient’s Primary Care Provider (PCP) via message in the electronic record to request that they consider a palliative care consult for the patient and order consultation if deemed appropriate. Therefore, the algorithm makes no clinical decisions; rather, the palliative care specialists leverage the algorithm for recommendations, and specialists and PCP together determine if palliative care is appropriate for each patient. Accepted patients in the control arm are not acted upon, but may continue to receive palliative care under the standard of care. Because of this, the trial has no blinding: palliative care and primary care specialists are informed which units are on which arms at what times.

**Fig. 2:**
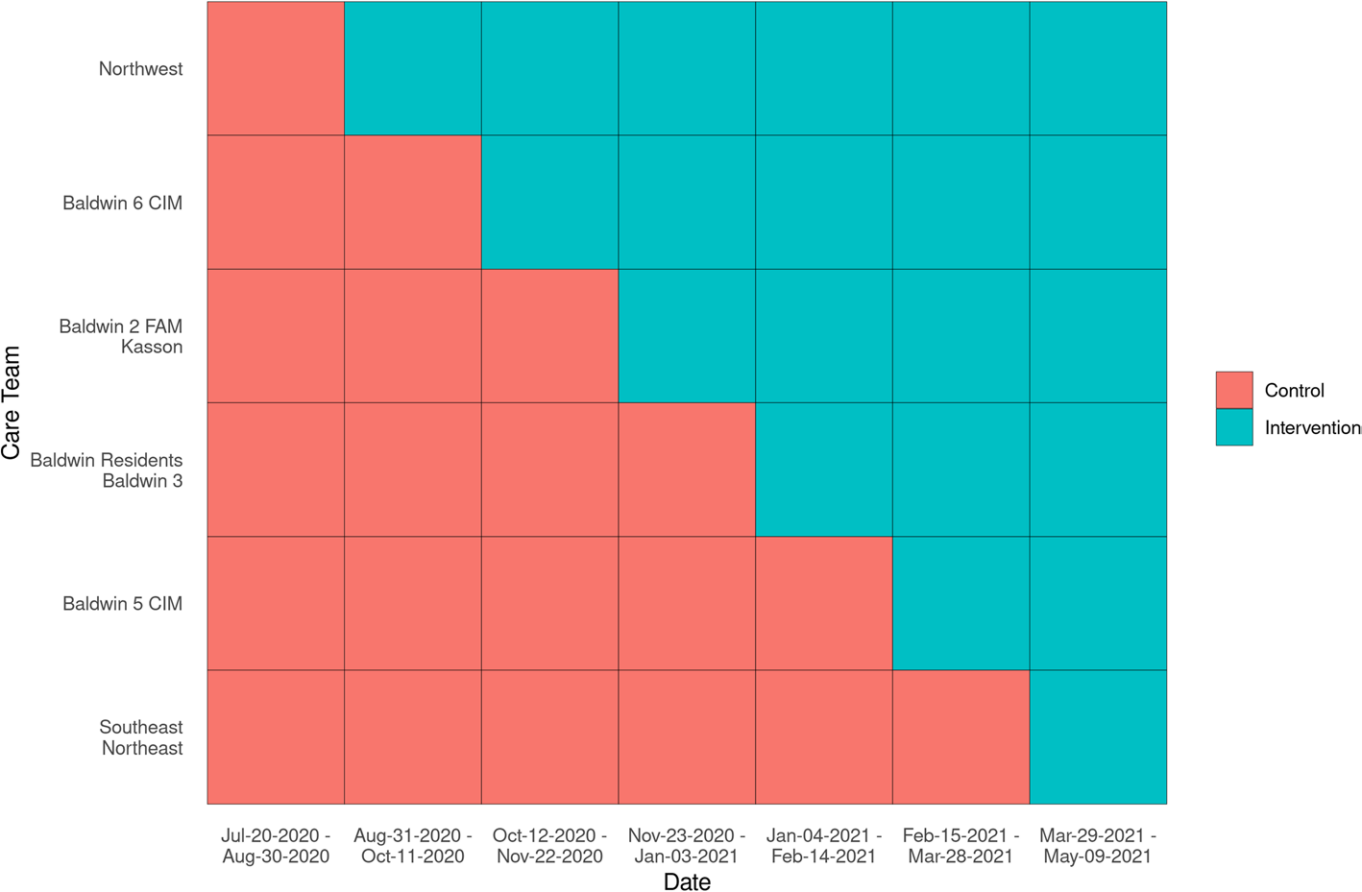
The stepped-wedge design matrix. Nine care teams were randomized into 7 wedges

Care team clusters were randomized (computer generated) into a seven-cluster stepped-wedge design matrix (Fig. 2). Several clusters were grouped together based on distribution of predicted palliative care score in historical data. New care teams will enter the treatment arm every 42 days. In this way, the first wedge sees all units on the control arm, and the last wedge sees all units on the treatment arm.

### Outcomes

The primary outcome is timely identification of palliative care consultation. This is measured as time to record of consult in the electronic health record by the palliative care team in the outpatient setting. Since the primary hypothesis was to reduce the time to palliative consult (in part by reducing the time to identification of palliative need), a time-to-event primary outcome was chosen over an outcome involving presence/absence of palliative consult. The secondary outcomes are:

1. Number of palliative care consultations over the study duration in each arm
2. Number of advanced care planning notes documented in the electronic health record in each arm
3. Number of billing codes (ICD-10) for palliative care in each arm
4. Positive predictive value (PPV) of screened patients: the percentage of patients presented for review who (1) are accepted by the palliative care specialists or, separately, (2) are accepted by both palliative care specialists and primary care
5. Performance metrics on palliative care specialist/PCP handoff: descriptive statistics on time between PCP contact and PCP response

Summarized patient data will be characterized by age, sex, and model probability as well as important static and time-varying covariates used in the algorithm. All patients will be analyzed on an intention-to-treat status regardless of whether they were accepted or not and will be extended to the cluster status in the event of care team change between intervention and control units.

### Analysis

For all study outcomes Bayesian hierarchical modeling will be used to adjust for the key features inherent in the stepped-wedge design. Particularly, time-to-event modeling will be used to model timely palliative care, the main study outcome, and other time-to-event or count outcomes. The time of event will consist of a heterogeneous Poisson process, allowing for adjustment to the event rate due to secular time effects and unit clustering through random effects. Unit clustering will be treated with normal random effects and the secular trend will be modeled with time series autoregressive prior of order 1. Statistical tests will be based on 95% credible intervals. For secondary binary outcomes, logistic regression will be used with the same design features.

### Power

**Fig. 3:**
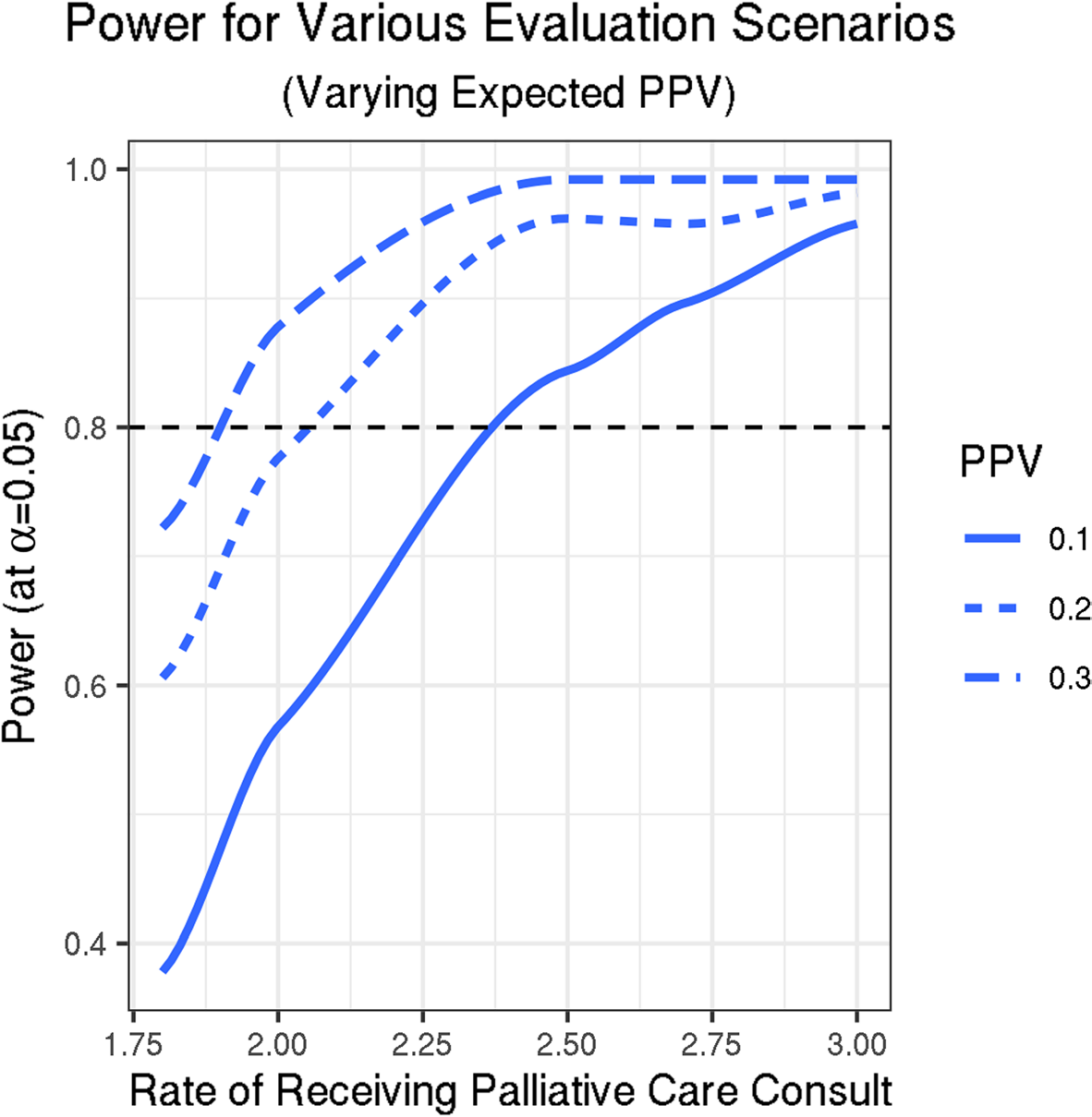
Power curves estimated using Monte Carlo simulation

To take account of the complex nature of the design, power was estimated using Monte Carlo simulation [11]. The model for the simulation consisted of a hierarchical Poisson regression with the outcome being time to palliative care. Random effects for cluster as well as a time series autoregressive model for secular trend were integrated to correctly specify the wedge design. To estimate reasonable parameters for this model pilot data were collected for all patients paneled to Mayo Clinic Primary Care teams as of September 2018 (n=154,312). For each patient, their palliative care consult status from the medical record and estimated probability from the algorithm were collected. With estimates of the intra-correlation of clusters (i.e., the rate of palliative care per care team), and secular trend estimated from data we have at least 80% power with reasonable PPVs in several scenarios for the trial timeframe to detect Incident Rate Ratios of 2.0 or greater with the stepped wedge design. See Fig. 3 for the power curves; various scenarios with varying number of clusters and time windows were tested.

### Approvals

All study activities were approved by the Mayo Clinic Institutional Review Board (ID 20-005977). Waivers of consent were granted for both patients and providers. Updates to the protocol are always communicated to the study team by email, and to participating care teams by a variety of electronic and in-person formats. Results of the study will be published as appropriate.

### Monitoring

Because there are minimal risks to this study, no formal monitoring committee was formed, nor will interim analyses be performed.

### Security/Privacy

All clinical data is pulled directly from the electronic health record, and is maintained on password-protected servers. Additionally, these data are stored in a secure MariaDB SQL database that the user interface accesses; both the user interface and the SQL database are password-protected and available only to palliative care specialists and statistical personnel.

## Discussion

Herein is described a pragmatic clinical trial to discern the effect of a machine learning algorithm on the timeliness of receipt of palliative care for patients in an outpatient primary care setting. Previous studies [7] have examined this relationship in the inpatient setting, but additional challenges are present in the outpatient setting. In particular, primary care providers may be difficult to engage, as they may have misperceptions about palliative care and less of a relationship with the palliative care specialty than inpatient physicians do.

Nevertheless, this trial retains the strengths of a pragmatic trial. Waivers of consent were obtained for both patients and providers, lessening the burden on patients and the clinical practice. This will make for easier integration of the algorithm into the practice. In addition, palliative care specialists will be reviewing the algorithm’s recommendations, lessening the number of false positives that reach the primary care physicians and hence any alarm fatigue. The specialists will also reach out to the primary care physicians directly, so referrals will come from a colleague instead of simply from an unfamiliar algorithm. Finally, the study endpoints are all available electronically as part of the electronic health record or the custom user interface.

One limitation of the pragmatic setup of this trial is the possibility of historical palliative consults being skewed toward certain populations or under-labeled in certain populations. Nevertheless, any subgroup of the patient population in which the algorithm experiences deficiencies will still experience palliative consults under the standard of care. For any subgroup toward which the label is skewed or more highly labeled, those patients will have their palliative needs met more effectively after being identified by the algorithm. Therefore, this study is still valuable to identify unmet palliative needs and because of its pragmatic nature can be easily implemented.

## Data Availability

Not applicable.

## Abbreviations

GBM: Gradient Boosting Machine
PCP: Primary Care Provider
PPV: Positive Predictive Value
QOL: Quality of Life
